# Unraveling the Electrochemical Insights of Cobalt Oxide/Conducting Polymer Hybrid Materials for Supercapacitor, Battery, and Supercapattery Applications

**DOI:** 10.3390/polym16202907

**Published:** 2024-10-15

**Authors:** Annu, Sang-Shin Park, Md Najib Alam, Manesh Yewale, Dong Kil Shin

**Affiliations:** School of Mechanical Engineering, Yeungnam University, 280 Daehak-ro, Gyeongsan 38541, Republic of Korea

**Keywords:** polypyrrole, polyaniline, conducting polymers, air batteries, pseudocapacitance, ion transport

## Abstract

This review article focuses on the potential of cobalt oxide composites with conducting polymers, particularly polypyrrole (PPy) and polyaniline (PANI), as advanced electrode materials for supercapacitors, batteries, and supercapatteries. Cobalt oxide, known for its high theoretical capacitance, is limited by poor conductivity and structural degradation during cycling. However, the integration of PPy and PANI has been proven to enhance the electrochemical performance through improved conductivity, increased pseudocapacitive effects, and enhanced structural integrity. This synergistic combination facilitates efficient charge transport and ion diffusion, resulting in improved cycling stability and energy storage capacity. Despite significant progress in synthesis techniques and composite design, challenges such as maintaining structural stability during prolonged cycling and scalability for mass production remain. This review highlights the synthesis methods, latest advancements, and electrochemical performance in cobalt oxide/PPy and cobalt oxide/PANI composites, emphasizing their potential to contribute to the development of next-generation energy storage devices. Further exploration into their application, especially in battery systems, is necessary to fully harness their capabilities and meet the increasing demands of energy storage technologies.

## 1. Introduction

**Figure 1:**
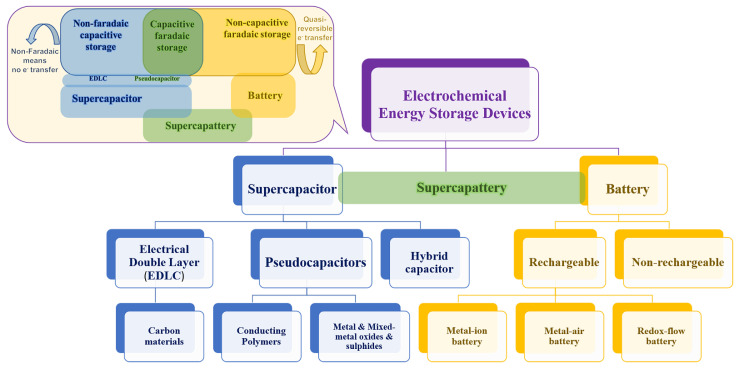
Hierarchical illustration of electrochemical energy storage devices.

In the quest to meet the ever-increasing global demand for energy, scientists and engineers are turning towards innovative energy storage solutions, particularly supercapacitors and batteries, due to their ability to deliver high power density and rapid energy release [1]. Unlike traditional capacitors and batteries, supercapacitors store and release electrical energy through electrostatic principles at the electrode–electrolyte interface, enabling them to store a significant amount of charge. This characteristic results in a high power density and rapid charge-discharge capability, making them suitable for applications requiring quick energy delivery [2]. Additionally, supercapacitors and batteries offer numerous advantages, including a wide operational temperature range, long cycle life, and high energy conversion efficiency, making them an attractive option for future energy storage technologies [1,2,3,4]. The basic hierarchy of electrochemical energy storage devices is presented in Figure 1, with the inset showing basic electrochemical characteristics of supercapacitors, batteries, and supercapatteries.

Transition metal oxide has a high energy density and better chemical stability [5]. The purpose of choosing cobalt oxide is mainly because of the recent advancements focused on cobalt oxide and its hybrid materials for electrochemical applications. While other transition metals such as nickel (Ni), iron (Fe), molybdenum (Mo), and manganese (Mn) also exhibit promising features, cobalt oxide (CoO, Co_3_O_4_) presents a series of advantages that justify its prioritization in this context. Table 1 shows a comparative discussion to substantiate the choice of cobalt oxide. Over the past five years, significant progress has been made in fabricating cobalt oxide nanostructures with various dimensionalities for applications in rechargeable batteries, supercapacitors, and electrocatalysis [6,7]. Cobalt oxide is gaining attention as a promising electrode material for next-generation electrochemical energy devices due to its favorable properties, such as low cost, abundance, high theoretical capacity and capacitance, excellent electrochemical activity, and robust chemical and mechanical stability. These characteristics make cobalt oxide an attractive alternative to other metal oxides in energy storage applications [8].

Sustainable methods have been developed recently to produce cobalt oxide nanoarchitectures. Raimundo et al. [9] explored the synthesis of a Co_3_O_4_-CoO nanocomposite using agar-agar through a two-step biogenic process for oxygen evolution reaction (OER) [10]. Natural lemon extract was employed for cobalt oxide nanoparticles (NPs) formation for an LPG gas sensor application [11]. Edison et al. [12] synthesized carbon-supported cobalt oxide NPs, which led to the best specific capacitance, which was calculated to be 642 Fg^−1^ at 1 Ag^−1^ in 2 M KOH solution. The green Co_3_O_4_@C NPs synthesized using *T. chebula* fruit shows good pseudocapacitance activity. The milk sap of *Calotropis procera* was also utilized for the synthesis of cobalt oxide NPs by the chemical growth method, and their supercapacitor application was reported by Bhatti et al. [13]. The supercapacitor efficiency of cobalt oxide NPs synthesized using marine red algae (*Grateloupia sparsa*) was recently investigated. The algae’s carbohydrates and polyphenols aided in reducing and stabilizing the NPs. The study found that the activated carbon/cobalt oxide nanocomposite had a significantly higher specific capacitance than raw activated carbon. Electrochemical analysis showed a specific capacitance of 125 Fg^−1^ for the cobalt oxide electrode in a 1M Na_2_SO_4_ electrolyte, with 93.75% capacity retention after 8000 cycles [14]. Huang et al. [15] investigated the supercapacitor performance of biogenic cobalt oxide NPs through *Bacillus pasteurii* bacteria in aqueous KOH by using the MIP (microbial-induced precipitation) process and showed a specific capacitance of about 162.78 Fg^−1^ at a current density of 1 mA cm^−2^ with a power density and an energy density as 64.29 W^.^ and 4.58 Wh·kg^−1^, respectively. Srivastava et al. [5] investigated the biogenic production of cobalt oxide NPs via *Citrus reticulata* (Mandarin oranges) fruit extract, and the specific capacitance was calculated to be 90 Fg^−1^ at 1 Ag^−1^ in electrolyte/solution. Shim et al. [16] proposed the synthesis of cobalt oxide NPs by using *Micrococcus lylae* bacteria and showed its supercapacitor performance. The electrochemical studies revealed a specific capacitance of 214 Fg^−1^ at 2 A g^−1^ for the cobalt oxide electrode, with a remarkable cyclic stability in aqueous electrolyte solution (3 M KOH) and 95% capacity retention after 4000 cycles at 5 mAcm^−2^ current density. All such studies prove that cobalt oxide not only has the ability to mitigate with minimal toxicity but is also significant in energy storage technologies.

**Table 1:** Comparison of electrochemical behavior of cobalt oxide and other transition metals.

Material	Specific Capacitance	Current Density/Scan Rate	Energy Density	Power Density	Ref.
Co_3_O_4_@Co(OH)_2_	1164 F g^−1^	1.2 A g^−1^	9.4 mWh cm^−3^	354 mW cm^−3^	[17]
Co-NiO@ carbon textile	106 F g^−1^	10 mA cm^−2^	52 Wh kg^−1^	1206 W kg^−1^	[18]
Co_2_O_4_@Zn-CuO	890 F g^−1^	1 A g^−1^	36 Wh kg^−1^	4800 W kg^−1^	[19]
CuO nanowires	594 F g^−1^	0.71 A g^−1^	35 Wh kg^−1^	520 W kg^−1^	[20]
CuO@Ni foam	431 F g^−1^	3.5 mA cm^−2^	19.7 Wh kg^−1^	7 kW kg^−1^	[21]
CuO nanoflowers	612 F g^−1^	1 A g^−1^	27 Wh kg^−1^	800 W kg^−1^	[22]
FeCo_2_O_4_	231 C g^−1^	1 A g^−1^	400 Wh kg^−1^	930 W kg^−1^	[23]
LaMnO_3_-NiCo_2_O_4_@NF	811 C g^−1^	0.5 A g^−1^	37 Wh kg^−1^	800 W kg^−1^	[24]
NiO flakes	574 F g^−1^	0.1 mA cm^−2^	11 Wh kg^−1^	124 W kg^−1^	[25]
NiCo_2_O_4_@MnMoO_4_	1118 F g^−1^	1 A g^−1^	237 Wh kg^−1^	700 W kg^−1^	[26]
ZnCo_2_O_4_@Ni(OH)_2_	4.6 F cm^−2^	2 mA cm^−2^	49 Wh kg^−1^	428 W kg^−1^	[27]
MoS_2_@Ti_3_C_2_Tx	1022.7 F/g	1 A g^−1^	54.7 Wh kg^−1^	1601.3 W kg^−1^	[28]
Bi_2_O_3_-Sb_2_O_4_-ZrO	441 F/g	1 A g^−1^	18 Wh/kg	-	[29]
MoS_2_/N-rGO	539.5 F g^−1^	1 A g^−1^	71.5 Wh kg^−1^	25.4 kW kg^−1^	[30]

However, the pristine forms of cobalt oxide still face challenges, such as poor electrical conductivity, slow reaction kinetics, and significant morphological and volume changes during electrochemical reactions. These intrinsic drawbacks often lead to rapid performance degradation, poor structural stability, limited cycling performance, and slow activation processes.

To overcome these limitations, researchers have been exploring cobalt oxide-based hybrids that combine the unique advantages of each component, resulting in synergistic effects that improve electronic conductivity, enhance reaction kinetics, and buffer volume changes during repeated charge-discharge cycles. These hybrid materials, compared to pristine cobalt oxide nanostructures, demonstrate enhanced rate capability, improved cycling stability, reduced overpotential, and overall better electrochemical properties, making them more suitable for energy-related applications. Despite these advances, challenges remain in optimizing cobalt oxide-based composites for practical electrochemical energy storage applications.

**Figure 2:**
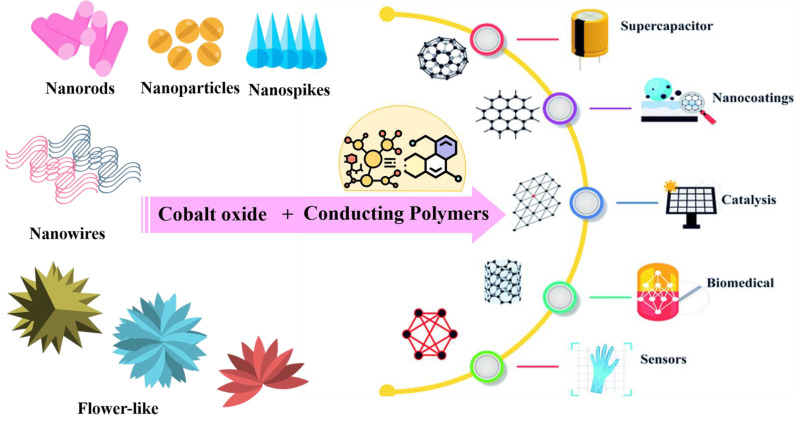
Representation of different morphologies of cobalt oxide for cobalt oxide/polymer nanocomposites and their possible applications [41].

Conducting polymers have also emerged as a significant class of materials in the development of supercapacitor electrodes. Among them, conductive polymers such as polyaniline (PANI) and polypyrrole (PPy) are frequently used to hybridize with metal oxides such as TiO_2_ and cobalt oxide nanostructures to enhance their electrical conductivity, improve adhesion, and facilitate rapid charge transfer [31,32]. The strategy to incorporate polymers in the electrode material assembly is sustainable compared to other metal incorporation. Besides, the storage mechanism of these conductive polymers primarily relies on redox reactions. During oxidation, ions are incorporated into the polymer backbone and subsequently released into the electrolyte during reduction, enabling efficient charge storage and release [33,34]. The integration of cobalt oxides with conductive polymers can greatly enhance the reaction rate and improve the ion-storage performance, resulting in superior electrochemical properties [35] (Figure 2).

Recent advancements in electrode materials for supercapacitors emphasize the synergy between multiple components to enhance electrochemical performance. Dongxian et al. synthesized a ternary composite (rGO/NiCo/PPy) with a sea urchin-like structure using a combination of Hummer’s and hydrothermal methods, followed by in-situ polymerization. This composite achieved a high specific capacitance of 333.2 F/g at 1 A/g and retained 94% capacitance after 7000 cycles. The assembled asymmetric supercapacitor displayed superior performance, reaching a specific capacitance of 253 F/g at a power density of 1250 W/kg [35].

Similarly, Ishaq et al. presented a one-step method to create binary (rGO/CoFe_2_O_4_) and ternary (rGO/CoFe_2_O_4_/PPy) nanocomposites. The ternary composite showed significantly enhanced performance, with a specific capacitance of 164 F/g and energy density of 22.8 Wh/kg, thanks to the conductive properties of PPy. This method offers advantages in terms of simplicity, cost-efficiency, and sustainability compared to more complex approaches [36].

Zhou et al. developed a supercapacitor electrode comprising CoO nanowires on 3D nickel foam with a PPy coating. This architecture leverages the high electrochemical activity of CoO and the conductivity of PPy, achieving a specific capacitance of 2223 F g^−1^, 99.8% retention after 2000 cycles, and high energy and power densities in an asymmetric supercapacitor device, which efficiently powered LEDs and a mini motor [37]. Nayak et al. used a chitosan biopolymer binder for supercapattery electrodes to create a silver-zirconia composite. This material demonstrated an energy density of 31.94 Wh/kg at a power density of 500.86 W/kg and retained 89% of its capacity after 2500 cycles at 10 A/g [38]. Among various conducting polymers, polyaniline (PANI) is the most extensively studied due to its variable oxidation states, which allow for tunable pseudocapacitive performance. Its high conductivity and ease of synthesis further add to its appeal. PANI has been widely used not only as a standalone supercapacitor electrode but also as a conductive additive to enhance the electrochemical performance of other materials. Recently, Kuchena and Wang demonstrated that an ammonium-ion battery cell utilizing an emeraldine salt (ES-PANI) cathode material achieved a commendable discharge capacity of 160 mAh g^−1^ at a current of 1 A g^−1^. Additionally, the battery exhibited strong capacity retention, maintaining 82% of its capacity after 100 cycles at a higher current of 5 A g^−1^, along with excellent rate capability [39]. As a result, it has been successfully integrated with cobalt oxides, mixed-metal oxides, carbon materials, and metal sulfides to develop advanced supercapacitor electrodes with enhanced performance. For instance, Hai et al. synthesized core–shell structured PANI-Co_3_O_4_ nanocomposites using a carbon-assisted in-situ polymerization method, achieving high specific capacitance and excellent cycling stability, with 84.9% capacity retention after 1000 charge-discharge cycles [40].

This review explores the sustainable perspectives of cobalt oxide/polymer nanocomposites for supercapacitor, battery, and supercapattery applications, highlighting the synthesis of electrode materials, their electrochemical performance associated with recent advancements, current challenges, and future directions in this rapidly evolving field. The amalgamation of cobalt oxides with conducting polymers, especially PPy and PANI, presents a promising avenue for developing high-performance, durable, and environmentally friendly energy storage devices, offering significant potential for meeting future energy demands. Moreover, we present focused data on green synthesized cobalt oxide, highlighting its potential to create sustainable electrode materials. This information aims to guide researchers in the development of environmentally friendly supercapacitors and batteries. The general electrochemical differences between supercapacitors, batteries, and supercapatteries are discussed in Table 2.

## 2. Cobalt Oxide/Polymer Nanocomposites

**Figure 3:**
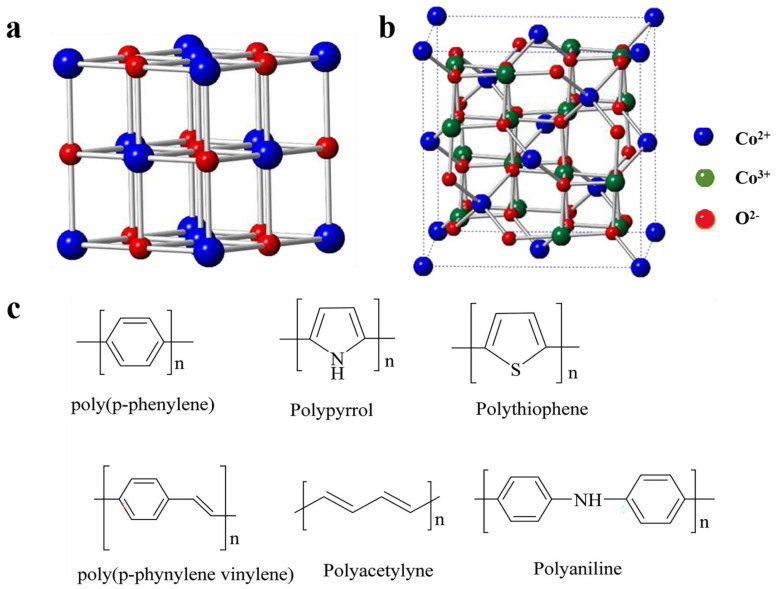
(**a**) Structure of cobalt oxide spinels CoO and (**b**) Co_3_O_4_ [8] and (**c**) different conducting polymers [41].

The integration of cobalt oxide with conducting polymers like polypyrrole (PPy) and polyaniline (PANI) has gained significant attention in the field of energy storage, particularly for supercapacitor applications. The combination of these materials leverages the high pseudocapacitance of cobalt oxide with the excellent conductivity and flexibility of conducting polymers, resulting in enhanced electrochemical performance. The crystal lattice structural representation of cobalt oxide spinels and different types of conducting polymers are depicted in Figure 3. This section delves into the synthesis methods, roles, and factors influencing the performance of cobalt oxide/PPy and cobalt oxide/PANI nanocomposites for supercapacitors.

## 3. Cobalt Oxide/PPy Nanocomposites for Supercapacitors

The cobalt oxide/PPy nanocomposites combine the high theoretical capacitance of cobalt oxide with the excellent electrical conductivity and flexibility of PPy, resulting in enhanced electrochemical performance beneficial for supercapacitors. Table 3 depicts the electrochemical properties of different cobalt oxide/PPy electrodes used in fabricating high-energy supercapacitors.

### 3.1. Synthesis of Cobalt Oxide/PPy Nanocomposites

Cobalt oxide/PPy nanocomposites are typically synthesized using methods that ensure uniform distribution of PPy over cobalt oxide nanostructures [42,43,44]. Common synthesis techniques include:

*In-situ polymerization*: In this method, pyrrole monomers are polymerized in the presence of pre-synthesized cobalt oxide NPs or nanostructures. The cobalt oxide acts as a template and provides a site for pyrrole polymerization, ensuring a uniform coating of PPy over the cobalt oxide surface.

*Electrochemical polymerization*: This technique involves the electrochemical deposition of PPy onto cobalt-oxide-coated electrodes. The electrochemical process facilitates the formation of a conformal PPy layer on cobalt oxide, enhancing the composite’s electrical conductivity and mechanical stability.

*Chemical oxidation polymerization*: In this method, cobalt oxide is mixed with pyrrole monomers in the presence of an oxidizing agent, leading to the polymerization of pyrrole and the formation of a cobalt oxide/PPy composite. This method allows for the fine tuning of the composite’s morphology by adjusting the concentration of the monomers and oxidizing agents.

### 3.2. Advantages of PPy in Supercapacitors

PPy enhances the performance of cobalt-oxide-based supercapacitors through several mechanisms, such as by increasing conductivity, pseudocapacitance, and structural stability. *Improved conductivity*: PPy is a highly conductive polymer that increases the overall electrical conductivity of the composite. This enhances the charge transfer between the electrode and the electrolyte, improving the rate capability and specific capacitance. *Pseudocapacitance contribution*: PPy contributes to the pseudocapacitance through its redox-active nature. The oxidation and reduction of PPy during charging and discharging cycles provide additional capacitance, complementing the pseudocapacitance of cobalt oxide. *Structural stability*: The flexibility of PPy helps in accommodating the volume changes of cobalt oxide during the charge-discharge cycles, thereby enhancing the cycling stability of the supercapacitor [43].

### 3.3. Electrochemical Performance of Cobalt Oxide/PPy Nanocomposites in Supercapacitors

*Electrochemical Mechanism*: Cobalt oxide/PPy nanocomposites exhibit enhanced electrochemical performance due to the synergistic effects of cobalt oxide and PPy. The electrochemical behavior of these nanocomposites is primarily governed by two key mechanisms:

*(i)*  *Faradaic redox reactions of cobalt oxide*: Cobalt oxide, particularly in its spinel form Co_3_O_4_, undergoes reversible redox reactions during the charging and discharging process. These reactions involve the oxidation and reduction of cobalt ions, contributing significantly to the pseudocapacitance. These reactions are facilitated by the high surface area of the nanostructured cobalt oxide, which provides a large number of active sites for redox reactions. The typical reactions are (Equations (1) and (2)):
  (1) $${Co}_{3}O_{4}+{OH}^{-}+H_{2}O\to 3\mathrm{CoOOH}+e^{-}$$
  (2) $$\mathrm{CoOOH}+{OH}^{-}\to {\mathrm{CoO}}_{2}+H_{2}O+e^{-}$$
*(ii)*  *Capacitive behavior of PPy*: PPy contributes to the overall capacitance of the nanocomposite through its capacitive properties, which arise from the doping and dedoping processes. During charging, PPy undergoes oxidation, where it accepts electrons, and during discharging, it is reduced, releasing electrons. This redox process is accompanied by the exchange of ions (usually Cl^−^ or SO_4_^2−^) between the PPy matrix and the electrolyte. The reaction can be represented as (Equation (3)):
  (3) $${\mathrm{PPy}}^{0}+{Cl}^{-}\to {\mathrm{PPy}}^{+}{Cl}^{-}+e^{-}$$

The high conductivity of PPy facilitates fast electron transfer, enhancing the overall charge-discharge rates and cyclic stability of the nanocomposite.

Recent studies focused on improving synthesis methods and structural design demonstrated the superior electrochemical performance of cobalt oxide/PPy nanocomposites to optimize their performance. For example, Ramesh et al. synthesized Co_3_O_4_/PPy@N-MWCNT via an ultrasonication-mediated solvothermal method, yielding impressive results for both supercapacitors and glucose sensors. The composite exhibited a capacitance of ∼872 F/g at 0.5 A/g with excellent cycling stability (96.8% retention after 10,000 cycles) alongside superior glucose-sensing performance. These studies highlight the potential of cobalt oxide/polymer composites in energy storage and sensing applications [45]. Zhou et al. synthesized a Co_3_O_4_/PPy nanocomposite that showed a specific capacitance of 2223 F g^−1^, approaching the theoretical value for supercapacitors. The enhanced performance was attributed to the well-aligned Co_3_O_4_ nanowire array on a 3D nickel foam substrate, which was uniformly coated with PPy. This architecture allowed for a short ion diffusion pathway and efficient electron transfer, resulting in a high power density of 5500 W kg^−1^ and an outstanding cycling stability with 99.8% capacitance retention after 2000 cycles [37].

**Table 3:** Electrochemical behavior of different cobalt oxide/PPy electrodes for energy storage applications.

Electrode Material	Enhancement	Morphology	Synthesis Method	Specific Capacitance (F g^−1^)	Application	Ref.
CoO/PPy	-	Nanowires	Hydrothermal + chemical polymerization	2223 at 1 mA cm^−2^	Supercapacitor	[37]
PPy/Co_3_O_4_/Carbon paper	Carbon paper	Composite	Hydrothermal + electrodeposition	398.4 at 1 A g^−1^	Supercapacitor	[42]
Co_3_O_4_@PPy/MWCNT	MWCNT	Composite	Chemical polymerization + hydrothermal	609 at 3 A g^−1^	Supercapacitor	[44]
Co_3_O_4_/PPy/MnO_2_	MnO_2_	Core–shell	Hydrothermal + chemical polymerization	780 at 0.5 A g^−1^	Supercapacitor	[46]
Co_3_O_4_/AuPPy	Au	Nanowires	Hydrothermal + chemical polymerization	2062 at 5 mA cm^−2^	Supercapacitor	[47]
AC//Co_3_O_4_/PPy/MnO_2_	Activated carbon, MnO_2_	Nanowires	Hydrothermal + electrodeposition	629 at 1.2 mA cm^−2^	Supercapacitor	[48]
NiCo_2_O_4_/PPy/Carbon textiles	Carbon textiles	Nanowires	Hydrothermal + chemical polymerization	2244.5 at 1 A g^−1^	Supercapacitor	[49]

In a study, a novel electrode material composed of NiCo_2_O_4_ nanowire arrays (NWAs) on carbon textiles with a PPy nanosphere shell layer was reported by Kong et al. to significantly enhance pseudocapacitive performance. The highly conductive nature of PPy, combined with the short ion transport channels within the ordered mesoporous NiCo_2_O_4_ nanowire arrays, along with the synergistic interaction between NiCo_2_O_4_ and PPy, contributed to an impressive specific capacitance of 2244 F g^−1^. The material also exhibited excellent rate capability and notable cycling stability. Specifically, the NiCo_2_O_4_/PPy electrode retained around 89.2% of its capacitance after 5000 cycles.

**Figure 4:**
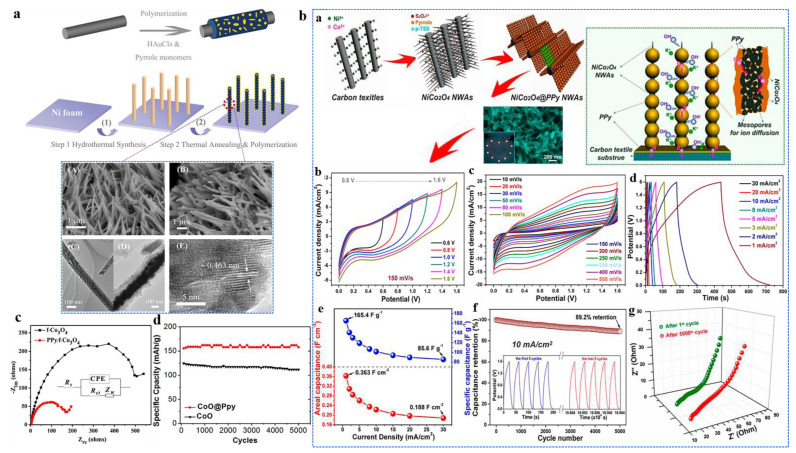
(**a**) Synthesis of cobalt oxide/PPy nanowires by hydrothermal followed by chemical polymerization [47]. (**b**) The charge transfer mechanism and electrochemical performance via CV curves at different potential windows (150 mV s^−1^) and different scan rates (0–1.6 V), GCD curves at different current densities, specific and volumetric capacitance and cycling performance of ASC device (10 mA cm^−2^) with Nyquist plots for the NiCo_2_O_4_/PPy/AC device (reprinted with permission from [49]; copyright 2015 American Chemical Society). (**c**) Nyquist plot of electropolymerized cobalt oxide/PPy/CP electrode [42] and (**d**) cycle stability of hydrothermally and electrodeposition polymerized CoO/Ppy nanoarrays [43].

Moreover, a lightweight and flexible asymmetric supercapacitor (ASC) was assembled using NiCo_2_O_4_/PPy NWAs as the positive electrode and activated carbon (AC) as the negative electrode. The ASC device demonstrated remarkable electrochemical performance, achieving a high energy density of 58.8 W h kg^−1^ at a power density of 365 W kg^−1^, as well as a peak power density of 10.2 kW kg^−1^ at an energy density of 28.4 W h kg^−1^, showcasing the material’s ability to deliver energy rapidly. Additionally, the cycling stability of the ASC device was excellent, retaining ∼89.2% of its initial capacitance after 5000 charge-discharge cycles in a gel electrolyte (KOH/PVA). In Figure 4a,b, a schematic illustration of the fabrication process for the hierarchical mesoporous Co/Au-PPy and NiCo_2_O_4_/PPy hybrid nanowires on carbon textiles is provided, highlighting the structure of the electrode material. Figure 4b(b,c) shows the cyclic voltammetry (CV) curves of the NiCo_2_O_4_/PPy NWAs and AC half cells measured in 3 M KOH solution at a scan rate of 10 mV s^−1^. It indicates that when the voltage is 1.0 V, two weak symmetric broad redox peaks can be observed, which indicate that the pseudocapacitive properties of the ASC device is derived from the positive electrode (NiCo_2_O_4_/PPy NWAs). It is evident that NiCo_2_O_4_/PPy NWAs exhibited superior electrochemical performance. Figure 4b(b) presents the CV curves of the NiCo_2_O_4_/PPy/AC ASC measured at different potential windows and a scan rate of 150 mV s^−1^. The results demonstrate the ASC’s wide operating voltage window and robust charge storage capabilities. The CV curves exhibited a quasi-rectangular shape, indicative of EDLC behavior, along with weak redox peaks, suggesting the presence of pseudocapacitive (PC) reactions. This combination of EDLC and PC contributions ensured that the device effectively stores energy. Even at a high scan rate of 500 mV s^−1^, the CV curve maintained its shape, demonstrating the good rate capability of the device, meaning it can handle fast charge-discharge cycles without significant degradation in performance. This retention of shape indicates strong electrochemical stability, even under rapid operation (Figure 4b(c)). Meanwhile, the charge-discharge profiles (Figure 4b(d)) as galvanostatic charge-discharge (GCD) curves were highly symmetric at all current densities, which signifies good Coulombic efficiency, meaning that the amount of charge stored during charging is almost equal to the amount of charge released during discharging. Even at the high current density of 30 mA cm^−2^, the device maintained excellent performance, with minimal voltage drop and well-preserved charge-discharge symmetry. This suggests that the ASC device exhibited outstanding electrochemical performance, fast charge transfer, and stable cycling behavior, even under high current stress, which is critical for applications requiring rapid energy storage and discharge. The specific and volumetric capacitance is depicted as a function of current density, showing the stable capacitance performance even at higher current densities (Figure 4b(e)). The cycling performance of the ASC devices, measured at a scan rate of 10 mA cm^−2^ over 5000 cycles, is depicted in Figure 4b(f), showing minimal capacitance degradation, as the inset compares the charge-discharge curves of the 1st and 5000th cycles as depicted in the Nyquist plots (Figure 4b(g)) for the ASC device, indicating low charge-transfer resistance, further confirming the device’s superior long-term electrochemical stability [49]. Similarly, the Nyquist plot and cycle stability of two different electrodes provided significant reliability in terms of their electrochemical advancements (Figure 4c,d).

### 3.4. Factors Influencing the Electrochemical Performance of Supercapacitors

Several factors influence the electrochemical performance of cobalt oxide/PPy nanocomposites and hence the supercapacitor, such as [42,43,44]:

*Morphology and porosity*: The nanostructure of cobalt oxide (e.g., nanowires, NPs, nanosheets) significantly affects the composite’s surface area and porosity, which are crucial for ion diffusion and charge storage. The morphology of the PPy coating also plays a role; a porous and uniform PPy layer facilitates efficient ion transport and enhances the electrode’s capacitance.

*Polymer content*: The amount of PPy relative to cobalt oxide is a critical parameter. An optimal PPy content maximizes conductivity and pseudocapacitance without compromising the active surface area of cobalt oxide. Excessive PPy can block active sites and reduce the overall capacitance.

*Electrode configuration*: The method of electrode fabrication, such as drop-casting or spin-coating of the composite onto current collectors, can affect the uniformity and adhesion of the composite layer, impacting the overall electrochemical performance.

## 4. Cobalt Oxide/PANI Nanocomposites for Supercapacitors

### 4.1. Synthesis of Cobalt Oxide/PANI Nanocomposites

**Figure 5:**
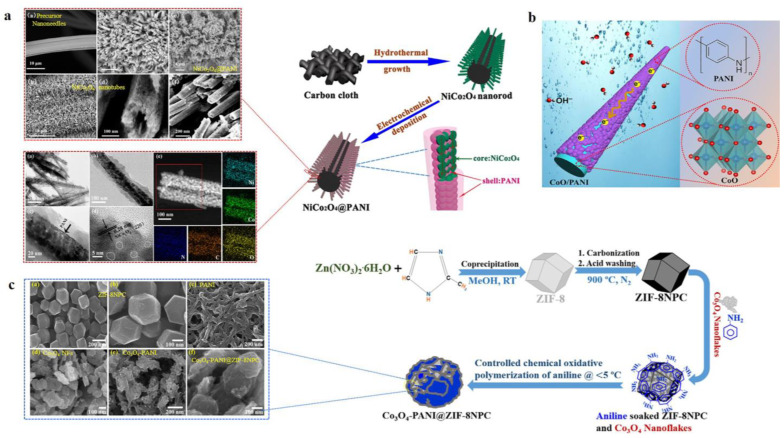
Synthesis methods: (**a**) hydrothermal followed by electrodeposition, (**b**) structural representation of CoO/PANI nanowires showing electron and electrolyte transport [53], and (**c**) in-situ chemical oxidative polymerization for NiCo_2_O_4_/PANI nanotubes [54] (reprinted with permission from [54]; copyright 2019 American Chemical Society), nanorods (reprinted with permission from [55]; copyright 2016 American Chemical Society), and a Co_3_O_4_-PANI/ZIF-8 nanoporous carbon (NPC) nanocomposite [56].

The synthesis of cobalt oxide/PANI nanocomposites generally involves techniques that promote strong interaction between polyaniline and cobalt oxide nanostructures. The common synthesis methods include [40,50,51]:

*In-situ chemical oxidation polymerization*: This method involves the polymerization of aniline monomers in the presence of cobalt oxide particles using an oxidizing agent. The process results in the formation of a uniform PANI coating on cobalt oxide, enhancing the composite’s electrical properties (Figure 5c).

*Hydrothermal synthesis followed by polymerization*: This is the most common method of fabricating electrode materials for supercapacitors. Cobalt oxide nanostructures are first synthesized via a hydrothermal method, followed by the oxidative polymerization of aniline (Figure 5a,b). This sequential approach allows for better control over the size and distribution of PANI on cobalt oxide

*Layer-by-layer assembly*: In this method, thin layers of cobalt oxide and PANI can be alternately deposited onto a substrate, creating a well-defined multilayer structure as done by Zhang et al. in the case of SnO_2_ nanosheets [52]. This technique allows for precise control over the thickness and composition of each layer, optimizing the electrochemical properties.

### 4.2. Advantages of PANI in Supercapacitors

PANI enhances the performance of cobalt-oxide-based supercapacitors by the following. *Increasing electrical conductivity*: PANI, like PPy, is a conductive polymer that significantly enhances the electrical conductivity of the composite, facilitating faster charge and discharge rates. *Enhancing pseudocapacitance*: PANI exhibits high pseudocapacitance due to its multiple oxidation states. The redox transitions of PANI contribute additional capacitance, complementing the faradaic reactions of cobalt oxide, thus improving the overall energy storage capacity. *Mechanical flexibility*: PANI’s flexibility helps to accommodate the volume changes of cobalt oxide during the charge-discharge cycles, thereby improving the durability and cycling stability of the composite electrode [31,51].

### 4.3. Electrochemical Performance of Cobalt Oxide/PANI Nanocomposites in Supercapacitors

*Electrochemical Mechanism*: Cobalt oxide/PANI nanocomposites demonstrate significant electrochemical performance enhancements due to the combination of the pseudocapacitive properties of cobalt oxide and the redox-active, conductive nature of PANI [40]. The electrochemical properties of different cobalt oxide/PANI electrodes for high-energy supercapacitors are depicted in Table 4.

*(i)*  *Faradaic redox reactions of cobalt oxide*: Similar to the cobalt oxide/PPy nanocomposite, the cobalt oxide in the cobalt oxide/PANI composite also undergoes reversible oxidation and reduction reactions. The redox reactions of Co^2+^/Co^3+^ and Co^3+^/Co^4+^ contribute to the overall pseudocapacitance. These reactions benefit from the high surface area provided by the nanostructured cobalt oxide, leading to enhanced redox activity. The reactions can be represented as (Equations (4) and (5)):
  (4) $${Co}_{3}O_{4}+{OH}^{-}+H_{2}O\to 3\mathrm{CoOOH}+e^{-}$$
  (5) $$\mathrm{CoOOH}+{OH}^{-}\to {\mathrm{CoO}}_{2}+H_{2}O+e^{-}$$
*(i)*  *Pseudocapacitive and conductive properties of PANI*: PANI contributes to the electrochemical performance through its pseudocapacitive properties, which are derived from its multiple redox states (leucoemeraldine, emeraldine, and pernigraniline). The redox transitions involve the transfer of protons (H⁺) and electrons (e^−^) and are represented as (Equation (6)):
  (6) $${PANI}^{0}+2H^{+}+2e^{-}\to {PANI}^{2+}(Emeraldine)$$

The ability of PANI to undergo such redox transitions at various oxidation states contributes to its high capacitance. Furthermore, the conductive backbone of PANI facilitates rapid electron transport, enhancing the overall charge storage and discharge capabilities of the nanocomposite.

Recent research has shown significant improvements in the electrochemical performance of cobalt oxide/PANI nanocomposites (Table 3). Hai et al. synthesized a core–shell structured Co_3_O_4_/PANI nanocomposite via a carbon-assisted in-situ polymerization method, achieving a high specific capacitance of 854 F g^−1^. The composite exhibited excellent cycling stability, with 84.9% retention of capacity after 1000 galvanostatic charge-discharge cycles. The improved performance was attributed to the unique core–shell architecture, which provided a large surface area for redox reactions and improved ion diffusion pathways [40].

**Figure 6:**
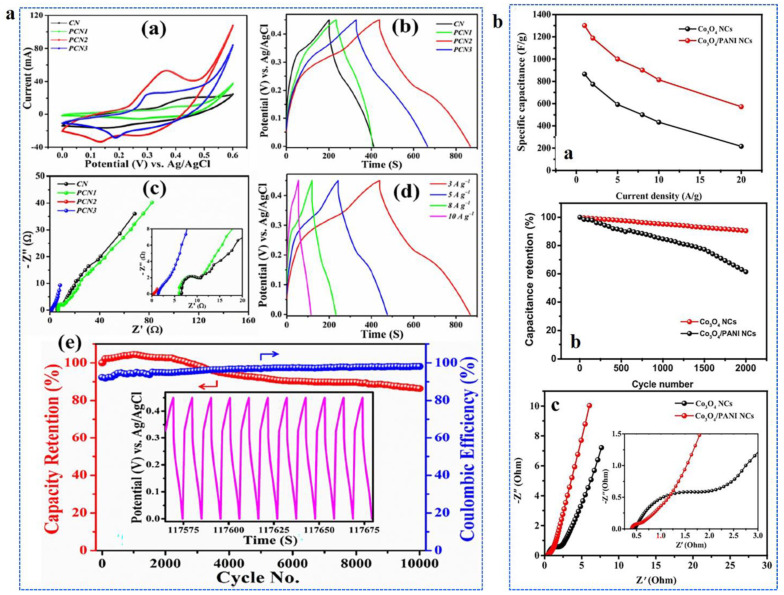
(**a**) Electrochemical performance of CoO/PANI nanowires: a—CV curves (scan rate 5 mV/s), b—charge-discharge profiles at 3 A/g, c—Nyquist plots, d—charge-discharge profiles at different current densities, and e—cycling stability curve of optimized PCN2, inset showing last few cycles [53]. (**b**) a—Specific capacitance, b—cycling stability at 10 A/g current density, and c—EIS spectra and Nyquist plot of Co_3_O_4_ and Co_3_O_4_/PANI nanocomposite, respectively [57].

Recently, a study on PANI/CoO nanowires grown on Ni foam (PCN) demonstrated a significantly enhanced electrochemical performance when used as a binder-free supercapacitor electrode. The core–shell structure of the PANI/CoO nanowires exhibited a high specific capacitance of 2473 F g^−1^ at a current density of 3 A g^−1^, with an excellent cycling stability of 86.3% after 10,000 charge-discharge cycles. The optimized design resulted in substantial synergistic effects between PANI and CoO, contributing to the high specific energy of 35.8 Wh kg^−1^, making the PCN2 electrode a highly promising candidate for future supercapacitors (Figure 6). Figure 6a(a) demonstrates that while the conductive emeraldine salt form of PANI was successfully produced in both PCN2 and PCN3, the capacitive performance of PCN3 was lower compared to PCN2. This reduction in performance is attributed to the excessive polymer coating in PCN3, which resulted in a thicker layer around the CoO nanowire arrays. The thicker polymer coating hindered the rate of ion transport, leading to a decreased specific capacitance (Figure 6a(b)). This is very important to be noted. The electrochemical analysis of PCN2 and PCN3 revealed distinct performance differences. PCN2 exhibited the lowest equivalent series resistance (ESR) at 0.39 Ω, with the ESR trend closely following the specific capacitance results (Figure 6a(c)). The lower ESR in PCN2 suggests improved conductivity, which facilitates efficient electron and charge transfer during the electrochemical process. This enhancement was further supported by the Nyquist plot in the low-frequency region, where PCN2 demonstrated a more vertical profile compared to other electrode materials, indicating faster electrode kinetics and reduced resistance. In Figure 6a(d), the nonlinear charge-discharge profiles of PCN2 at different current densities point to the occurrence of faradaic reactions, confirming the electrochemical activity of the material during cycling. This behavior is indicative of the faradaic process driving the charge storage mechanism in PCN2. Figure 6a(e) shows a significant improvement in cycling stability, with the coulombic efficiency increasing from 92.3% to 98.1% over 10,000 cycles. This remarkable cycling stability can be attributed to the porous structure of the electrode, which allowed for better accommodation of the volume changes during charge-discharge cycling. Additionally, the presence of PANI helped to mitigate volumetric expansion and compression, ensuring that the electrode maintained structural integrity throughout prolonged cycling. As a result, PCN2 outperformed other Co-based electrodes in terms of long-term stability and electrochemical performance. Moreover, an asymmetric supercapacitor based on these PANI/CoO nanowire arrays demonstrated the capability to power 19 red LEDs [53]. Similarly, the ZIF-67 templates with the Co_3_O_4_/PANI nanocomposite synthesized by hydrothermal and chemical in-situ polymerization showed high specific capacitance (Figure 6b(a)) attributing to the high surface area and the short transport path for ions and electrons due to the hierarchically hollow structured Co_3_O_4_ and the highly conductive PANI layer. Moreover, the study revealed good cycle stability with 90% retention after 2000 cycles (Figure 6b(b)), which indicates the ability of such polymeric hybrid electrodes in enhancing the conductivity, pseudocapacitance, and stability (Figure 6b(c)) [57].

Chhetri et al. reported an advanced composite material for supercapacitors combining Zeolitic Imidazolate Framework-8 (ZIF-8)-derived nanoporous carbons (NPCs) with Co_3_O_4_ nanoflakes and PANI. The Co_3_O_4_-PANI@ZIF-8NPC nanocomposite was synthesized through controlled in-situ polymerization of PANI on the surface of ZIF-8NPC, with Co_3_O_4_ nanoflakes providing additional pseudocapacitive behavior. This unique nanostructure yielded a specific capacitance of 1407 F g^−1^ at a current density of 1 A g^−1^, demonstrating both remarkable rate capability and cyclic stability. The enhanced electrochemical performance was attributed to the combination of the high conductivity provided by PANI, the increased number of redox-active sites from Co_3_O_4_, and the high surface area and suitable pore size distribution of ZIF-8NPC. Furthermore, an ASC was assembled using Co_3_O_4_-PANI@ZIF-8NPC as the positive electrode and ZIF-8NPC as the negative electrode. This ASC exhibited outstanding energy storage capabilities, achieving energy densities of 52.81 Wh kg^−1^ at a power density of 751.51 W kg^−1^ and 18.75 Wh kg^−1^ at a power density of 7500 W kg^−1^. The ASC also demonstrated excellent cycling stability, retaining 88.43% of its initial capacitance after 10,000 cycles at a high current density of 10 A g^−1^ [56].

Another study by Jabeen et al. focused on addressing the structural instability of PANI, which often limits its cycle life. The researchers developed a NiCo_2_O_4_@PANI core–shell nanorod array as an electrode material for supercapacitors. This hybrid structure, with highly porous NiCo_2_O_4_ serving as the conductive core and PANI as the active pseudocapacitive material, achieved a high specific capacitance of 901 F g^−1^ at 1 A g^−1^ in 1 M H_2_SO_4_. Notably, the electrode demonstrated excellent cycling stability with a capacitance retention of 91% after 3000 cycles at 10 A g^−1^, which is a significant improvement compared to most reported PANI-based pseudocapacitors [55].

Similarly, NiCo_2_O_4_ has emerged as a promising supercapacitor material, though it requires improvement in capacitance and cycle stability for practical use. A NiCo_2_O_4_@PANI nanotube array on carbon textiles was synthesized via a two-step process, which resulted in a specific capacity of 720.5 C g^−1^ at 1 A g^−1^. The introduction of a PANI layer significantly improved the cycle stability, with 99.64% capacity retention after 10,000 cycles. The PANI coating not only provided additional pseudocapacitance but also protected the tubular NiCo_2_O_4_ structure from deformation, further enhancing its electrochemical performance. Detailed analyses revealed reduced charge transfer resistance and faster charge storage kinetics, indicating the superior performance of the NiCo_2_O_4_@PANI composite as a supercapacitor electrode [54].

Rajkumar et al. also reported the synthesis of FeCo_2_O_4_/PANI nanocomposites via an in-situ polymerization method. The resulting material exhibited an impressive specific capacity of 940 C g^−1^ at 1 A g^−1^, as measured by galvanostatic charge-discharge tests. The enhanced capacitive behavior was attributed to the nanorod-like structure of the composite, which provided a large surface area and a significant number of active sites, facilitating efficient ion and electron transport [58].

A recent study reported the synthesis of a ternary composite consisting of PANI, Co_3_O_4_ nanoparticles, and chitosan via an in-situ chemical oxidation method. The Co_3_O_4_ nanoparticles were uniformly coated with layers of chitosan and PANI, forming a core/double-shell nanostructure. The ternary composite demonstrated significantly improved specific capacitance compared to binary PANI/Co_3_O_4_ or PANI/CS composites. The optimal nanocomposite, with 40% Co_3_O_4_ nanoparticles, achieved a specific capacitance of 687 F g^−1^, along with an energy density of 95.42 Wh kg^−1^ at 1 A g^−1^ and a power density of 1549 W kg^−1^ at 3 A g^−1^. Additionally, the composite exhibited excellent cycling stability, retaining 91% of its capacitance after 5000 charge-discharge cycles. The synergistic effect between PANI, Co_3_O_4_, and CS contributed to the enhanced electrochemical performance [59].

These studies collectively demonstrate the effectiveness of incorporating PANI with metal oxides to enhance the electrochemical performance of supercapacitors. The synergistic effects between the metal oxides and PANI contribute to improved capacitance, cycling stability, and rate capability, making these nanocomposites highly promising candidates for advanced energy storage systems.

### 4.4. Factors Influencing the Electrochemical Performance of Supercapacitors

The performance of cobalt oxide/PANI nanocomposites is affected by several factors:

*Morphology and nanostructure*: The shape and size of cobalt oxide nanostructures (e.g., NPs, nanoflakes) determine the surface area and active sites available for redox reactions. The morphology of PANI, whether it forms a fibrous, tubular, or granular structure, influences the ion diffusion pathways and overall capacitance.

*PANI loading and distribution*: The amount and distribution of PANI in the composite are crucial for balancing conductivity and capacitance. Adequate PANI content ensures high conductivity and pseudocapacitance, but excess PANI can lead to a decrease in the active surface area and hinder ion transport.

*Porosity and surface area*: A high surface area and porosity of the composite facilitate better electrolyte penetration and ion diffusion, which are crucial for achieving high capacitance and fast charge-discharge cycles. The porosity of both the cobalt oxide and the PANI layers needs to be optimized for maximum performance.

**Table 4:** Electrochemical behavior of different cobalt oxide/PANI electrodes for energy storage applications.

Electrode Material	Enhancement	Morphology	Synthesis Method	Specific Capacitance (F g^−1^)	Application	Ref.
PANI-Co_3_O_4_	-	Core–shell	Hydrothermal + chemical polymerization	1184 at 1.25 A g^−1^	Supercapacitor	[40]
PANI/CoO/NF	Ni foam	Nanowires	Hydrothermal + electrodeposition	2473 at 3 A g^−1^	Supercapacitor	[53]
NiCo_2_O_4_/PANI carbon textiles	Carbon textiles	Nanotubes	Hydrothermal + chemical polymerization	720 C g^−1^	Supercapacitor	[54]
NiCo_2_O_4_/PANI/CC	Ni, Carbon cloth	Nanorods	Hydrothermal + electrodeposition	901 at 1 A g^−1^	Supercapacitor	[55]
carbon, Co_3_O_4_, and PANI	Carbon	Nanocomposite	Hydrothermal + Chemical oxidative polymerization	1407 at 1 A g^−1^	Supercapacitor	[56]
Graphene/PANI/Co_3_O_4_	Graphene	Nanocomposite	Chemical polymerization + hydrothermal	789.7 at 1 A g^−1^	Supercapacitor	[60]
FeCo_2_O_4_ and PANI	Fe	Nanorods	Hydrothermal + Chemical oxidative polymerization	940 C g^−1^	Supercapacitor	[58]
Co^2+^ doped PANI	-	Films	Electrodeposition	736 at 3 mA cm^−2^	Supercapacitor	[61]
Graphene/PANI/Co_3_O_4_ aerogel	Graphene	Aerogels	Chemical polymerization + hydrothermal	1247 at 1 A g^−1^	Supercapacitor	[62]
PANI/Co_3_O_4_/CS	Chitosan	Nanocomposite	In-situ chemical oxidation polymerization	687 at 1 A g^−1^	Supercapacitor	[59]
PANI/CoO/ZT	Zeolite	Composite	Co-precipitation + Chemical oxidative polymerization	1282 at 1 A g^−1^	Supercapacitor	[63]
Co_3_O_4_/PANI	ITO	Nanoshrubs	Sol-gel + Emulsion polymerization	1151 at 3 A g^−1^	Supercapacitor	[64]
Co_3_O_4_/PANI	ZIF-67	Nanocage	Precipitation + Chemical polymerization	1301 at 1 A g^−1^	Supercapacitor	[57]

## 5. Cobalt Oxide/PPy Nanocomposites for Batteries

The application of cobalt oxide/conducting polymer nanocomposites as electrode materials in batteries remains largely underexplored compared to their use in supercapacitors and hybrid supercapatteries. We have come across only a limited number of studies of cobalt oxide/PPy and cobalt oxide/PANI for battery applications. While these materials have shown promising electrochemical performance, further research is needed to fully understand their potential in battery applications. Expanding studies in this area will help optimize their structural, electrochemical, and cycling properties, paving the way for broader utilization in advanced battery technologies. The electrochemical performance of cobalt oxide-based nanocomposites combined with conducting polymers like PPy and PANI extends beyond supercapacitors to rechargeable battery applications, including lithium-ion batteries (LIBs), sodium-ion batteries (SIBs), and other emerging air battery systems [65].

### 5.1. Synthesis Strategies of Cobalt Oxide/PPy Nanocomposites for Batteries

Focusing on achieving uniform distribution, optimal morphology, and enhanced interface interactions between cobalt oxide and PPy, several synthesis methods have been employed to fabricate cobalt oxide/PPy nanocomposites. Common synthesis strategies include [66,67]:

*In-situ polymerization*: In this method, cobalt oxide NPs are first synthesized through a hydrothermal or sol-gel method. The pyrrole monomer is then added to the Cobalt oxide dispersion, and polymerization is initiated using an oxidizing agent (e.g., ammonium persulfate) under controlled conditions. This process allows PPy to form uniformly on the surface of cobalt oxide NPs, ensuring intimate contact and a robust composite structure.

*Electrochemical polymerization*: This method involves depositing cobalt oxide onto a substrate followed by electrochemical polymerization of pyrrole in a suitable electrolyte solution. The applied potential drives the polymerization, forming a PPy coating on the cobalt oxide NPs. This technique offers precise control over the thickness and uniformity of the PPy layer, which is crucial for optimizing electrochemical performance.

*Solvothermal and co-precipitation methods*: Cobalt oxide NPs are synthesized via solvothermal methods, followed by the addition of a pyrrole monomer. Polymerization is induced by temperature or chemical initiators. Coprecipitation of cobalt precursors with pyrrole ensures simultaneous formation of cobalt oxide and PPy, leading to a homogeneous nanocomposite structure.

### 5.2. Role of PPy in Enhancing Battery Performance

Similar to supercapacitors, in the case of batteries, PPy plays a critical role in improving the electrochemical performance of cobalt-oxide-based batteries with [4,65]:

*Enhanced conductivity*: PPy is an intrinsically conductive polymer that forms a continuous conductive network within the composite. This network facilitates efficient electron transport, which is vital for high-rate charge and discharge processes in batteries.

*Buffering effect*: PPy provides a flexible matrix that buffers the volumetric expansion and contraction of Cobalt oxide during the lithiation/delithiation or sodiation/desodiation cycles, thereby enhancing the structural stability of the electrode.

*Additional pseudocapacitive contribution*: PPy contributes to the overall capacity through its redox-active sites, which can store and release charge, thereby improving the specific capacity of the electrode.

### 5.3. Electrochemical Performance of Cobalt Oxide/PPy Nanocomposites in Batteries

*Electrochemical Mechanism*: Cobalt oxide is a well-known anode material for lithium-ion and sodium-ion batteries due to its high theoretical capacity and multiple oxidation states that facilitate reversible redox reactions. The integration of PPy enhances the electrochemical performance through the following mechanisms [66,67,68]:

*(i)*  *Lithium-ion batteries (LIBs)*: In LIBs, cobalt oxide undergoes conversion and alloying reactions during lithiation and delithiation processes. The electrochemical reactions can be summarized as in Equations (7) and (8):
  (7) $${Co}_{3}O_{4}+8{Li}^{+}+8e^{-}\to 3Co+4{Li}_{2}O$$

The reaction involves the conversion of cobalt oxide to metallic Co and Li_2_O during lithiation, and the reverse reaction occurs during delithiation. The Co NPs formed during this process act as active sites for the further reduction of lithium ions, contributing to the high capacity of the electrode.

*Role of PPy*: PPy serves as a conductive matrix, enhancing the electronic conductivity of the composite and buffering the volume changes associated with the redox reactions of cobalt oxide. PPy also provides a pathway for rapid electron transport, which is crucial for maintaining the structural integrity and high rate capability of the electrode.

*(ii)*  *Sodium-ion batteries (Na-ion batteries)*:

*Cobalt oxide redox reactions*: The electrochemical behavior in Na-ion batteries is similar to that in Li-ion batteries, with the conversion of cobalt oxide into Na_2_O and Co during the sodiation process:

(8) $${Co}_{3}O_{4}+8{Na}^{+}+8e^{-}\to 3Co+4{Na}_{2}O$$

*Role of PPy*: PPy enhances the sodium storage capacity by providing a conductive network that supports the redox reactions of cobalt oxide. Moreover, the flexible nature of PPy accommodates the strain from the volume changes during sodiation and desodiation, improving cycling stability.

Recent studies have explored novel nanocomposites involving PPy and cobalt-based compounds for advanced energy storage applications, including lithium-ion and sodium-ion batteries. Guo et al. synthesized a polypyrrole–cobalt–oxygen (PPy-Co-O) coordination complex, which demonstrated excellent lithium storage capacity and stability. Using extended X-ray absorption fine structure (EXAFS) and Raman spectroscopy, they revealed that this complex form when PPy-coated Co_3_O_4_ is cycled between 0.0 V and 3.0 V versus Li^+^/Li. DFT calculations suggested that each cobalt atom coordinates with two nitrogen atoms in the PPy-Co structure, while oxygen atoms link the layers. This coordination weakens C-H bonds in PPy, resulting in a high-capacity, long-lasting lithium-storage material [68].

**Figure 7:**
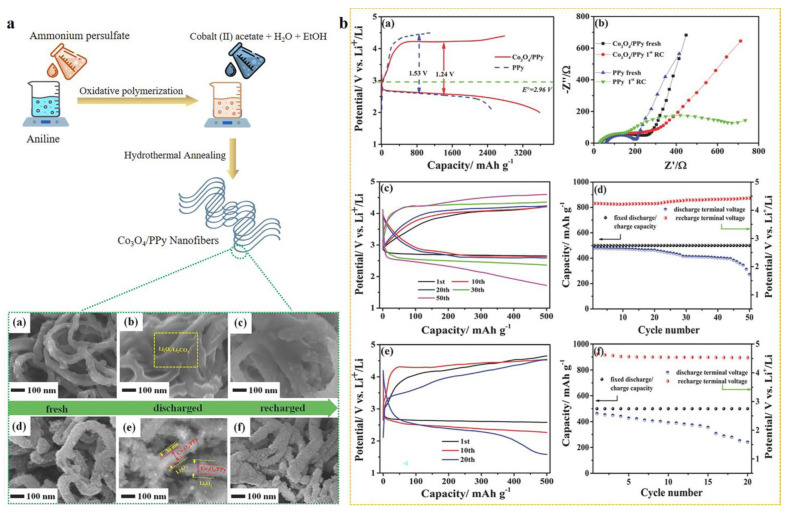
(**a**) Preparation method of Co_3_O_4_/PPy nanohybrid for batteries showing their SEM images. (**b**) Electrochemical performance of Co_3_O_4_/PPy nanofibers for LiO_2_ battery applications [69].

Similarly, Hou et al. developed a Co_3_O_4_/PPy nanohybrid through a hydrothermal process, optimized for bifunctional catalysis in lithium-oxygen (Li-O_2_) batteries (Figure 7a). The Co_3_O_4_/PPy hybrid material enhanced both oxygen reduction reaction (ORR) and oxygen evolution reaction (OER) performance, yielding a discharge capacity of 3585 mAh g^−1^ and a charge capacity of 2784 mAh g^−1^ at 100 mA g^−1^. The improved electrochemical performance was attributed to the synergy between the conductive PPy nanoweb structure and the high catalytic activity of Co_3_O_4_ nanoparticles [69]. Figure 7b demonstrates the enhanced electrochemical performance of the Co_3_O_4_/PPy hybrid due to synergistic effects between Co_3_O_4_ and PPy, contributing to its promising application in lithium-oxygen batteries. The study revealed the initial charge-discharge profiles of the nanofibrous Co_3_O_4_/PPy hybrid and pristine PPy cathodes in lithium-oxygen batteries. These curves were recorded at a current density of 100 mA g^−1^ in a 1 M LiCF_3_SO_3_/TEGDME electrolyte (operating between 2.0 and 4.4 V vs. Li^+^/Li) (Figure 7b(a)). The distinct profiles highlighted the superior capacity and energy efficiency of the Co_3_O_4_/PPy hybrid compared to the pristine PPy. The electrochemical impedance spectroscopy (EIS) plots compared the impedance of the Co_3_O_4_/PPy hybrid and the pristine PPy cathodes before and after the first recharge. The lower impedance of the Co_3_O_4_/PPy hybrid indicated better charge transfer kinetics and reduced resistance (Figure 7b(b)). Figure 7b(c,d) shows the charge-discharge curves and cycling performance of the Co_3_O_4_/PPy hybrid electrode, tested at a capacity limit of 500 mAh g^−1^ and a current density of 100 mA g^−1^, indicating stable cycling over multiple cycles. The performance highlights the hybrid’s ability to sustain capacity over prolonged use. Furthermore, the charge-discharge profiles and cycling performance of the pristine PPy cathode under the same testing conditions revealed a rapid decline in capacity with increasing cycle number. This suggests the superior stability and cycling retention of the Co_3_O_4_/PPy hybrid compared to the pristine PPy (Figure 7b(e,f)) [69].

In a recent study, cobalt-based binary sulfides were investigated for sodium-ion batteries (SIBs). The high abundance and cost-effectiveness of sodium make SIBs an attractive alternative for energy storage. However, challenges such as high-volume expansion and limited cycle life persist. A novel PPy/ZnCoS@CC nanocomposite, where zinc cobalt sulfide nanosheets were coated with PPy, was synthesized via a hydrothermal process. The PPy coating helped mitigate volume expansion and enhanced cycling stability, achieving an aerial capacity of 2.33 mAh cm^−2^, with 75% retention after 100 cycles. The study also utilized the galvanic intermittent titration technique (GITT) to measure the diffusion coefficient, showing that the conductive polymer coating played a crucial role in stabilizing the electrode during charge-discharge cycles [70].

These findings underscore the potential of PPy/cobalt nanocomposites for enhancing energy storage devices by addressing key limitations such as cycle stability, volume expansion, and electrochemical performance.

### 5.4. Factors Influencing the Electrochemical Performance of Batteries

The cobalt oxide/PPy nanocomposites used in batteries can be affected by different factors, which in turn influence the electrochemical performance of the batteries [4], such as:

*Morphology and porosity*: The porous structure of cobalt oxide/PPy nanocomposites provides a larger surface area for electrochemical reactions and facilitates ion diffusion, enhancing the battery’s capacity and rate capability.

*Polymer content*: The ratio of PPy to cobalt oxide is crucial; excessive PPy may lead to a reduction in specific capacity due to its lower theoretical capacity compared to cobalt oxide, while insufficient PPy might not provide adequate conductivity or structural support.

*Particle size distribution*: Uniform distribution of cobalt oxide particles within the PPy matrix ensures consistent electrochemical performance and prevents agglomeration, which can degrade the battery’s capacity and stability.

## 6. Cobalt Oxide/PANI Nanocomposites for Batteries

### 6.1. Synthesis Strategies of Cobalt Oxide/PANI Nanocomposites for Batteries

The synthesis of cobalt oxide/PANI nanocomposites involves similar strategies to those used for cobalt oxide/PPy composites, with a focus on achieving optimal polymerization and composite formation [71,72]:

*In-situ oxidative polymerization*: This method involves dispersing cobalt oxide NPs in an aniline monomer solution, followed by the addition of an oxidizing agent (e.g., ammonium persulfate). Polymerization occurs on the surface of cobalt oxide, forming a PANI coating. This method ensures strong interfacial bonding and uniform distribution of PANI on cobalt oxide.

*Template-assisted synthesis*: Cobalt oxide is synthesized using a template that provides a specific morphology (e.g., nanorods, nanowires). Aniline is then polymerized on the cobalt oxide template, producing a PANI-coated cobalt oxide nanocomposite. This method allows control over the morphology and surface area, which are crucial for enhancing electrochemical performance.

*Electrospinning and chemical vapor deposition (CVD)*: Electrospinning can be used to create a fibrous network of cobalt oxide embedded within a PANI matrix, providing a high surface area and porous structure. Alternatively, CVD allows for the precise deposition of PANI onto cobalt oxide NPs, enhancing uniformity and composite stability.

### 6.2. Role of PANI in Enhancing Battery Performance

PANI enhances the performance of cobalt-oxide-based batteries through several mechanisms:

*Improved electrical conductivity*: PANI is a highly conductive polymer that forms a continuous pathway for electron transport, reducing internal resistance and enhancing charge-discharge rates.

*Pseudocapacitive behavior*: PANI provides additional pseudocapacitive storage through its redox transitions (emeraldine to leucoemeraldine and pernigraniline states), which contribute to the overall charge storage capacity of the electrode.

*Mechanical flexibility and stability*: The flexible nature of PANI helps to accommodate the volumetric changes in cobalt oxide during cycling, reducing mechanical stress and improving the cycling stability of the composite.

### 6.3. Electrochemical Performance of Cobalt Oxide/PANI Nanocomposites in Batteries

*Electrochemical Mechanism*: The integration of cobalt oxide with PANI in the most common battery electrodes leads to enhanced electrochemical performance due to the following mechanisms:

*(i)*  *Lithium-ion batteries (LIBs):*

*Cobalt oxide (Co_3_O_4_) redox reactions*: Similar to the cobalt oxide/PPy system, cobalt oxide in cobalt oxide PANI nanocomposites undergoes a conversion reaction where cobalt oxide is reduced to Co and Li_2_O during lithiation. The delithiation process involves the oxidation of Co and the release of Li^+^ (Equation (9)).

(9) $${Co}_{3}O_{4}+8{Na}^{+}+8e^{-}\to 3Co+4{Na}_{2}O$$

*Reaction with PANI*: PANI not only enhances the electronic conductivity but also provides pseudocapacitive contributions from its redox-active sites. PANI can undergo reversible redox transitions between its leucoemeraldine, emeraldine, and pernigraniline states, contributing to the overall capacity. These transitions allow for additional charge storage and improve the rate capability of the electrode (Equation (10)).

(10) $${PANI}^{0}+2{Li}^{+}+2e^{-}\to {PANI}^{2+}(Emeraldine)$$

*(ii)*  *Sodium-ion batteries (Na-ion batteries):*

*Cobalt oxide redox reactions*: In Na-ion batteries, Co_3_O_4_ undergoes a similar conversion reaction as in Li-ion batteries (Equation (11)):

(11) $${Co}_{3}O_{4}+8{Na}^{+}+8e^{-}\to 3Co+4{Na}_{2}O$$

*Reaction with PANI*: PANI enhances the sodium storage capacity by providing a conductive network and additional pseudocapacitance from its redox-active sites. The flexibility of PANI also helps to accommodate the volume expansion associated with sodium insertion, which is more pronounced than lithium insertion, thereby improving the cycling stability.

**Figure 8:**
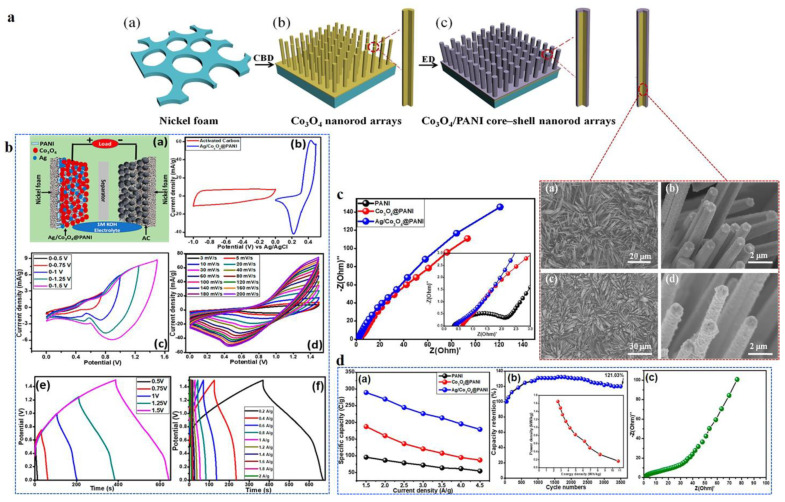
(**a**) Synthesis of Co_3_O_4_/PANI nanorods as an anode material by chemical bath deposition (CBD) followed by electrodeposition (ED) and its impedance spectra for Li-ion batteries [73]. (**b**) Supercapattery device fabrication by Ag/Co_3_O_4_/PANI nanocomposites as battery-type positive electrode material, showing its CV curves at different potential and scan rates, and charge-discharge at different potentials and current densities. Comparative analysis by (**c**) Nyquist plot and (**d**) specific capacity and current density of Ag/Co_3_O_4_/PANI nanocomposite along with the cycle stability and energy and power densities of the fabricated device [71].

Recently, Kuchena and Wang synthesized an ammonium-ion battery cell utilizing an emeraldine salt (ES-PANI) cathode material that exhibited a high discharge capacity of 160 mAh g^−1^ at 1 A g^−1^ in Li-ion batteries. The composite showed excellent rate capability, with a capacity retention of 82% after 100 cycles at 5 A g^−1^. The improved performance was attributed to the synergistic effects of ES-PANI, which enhanced electron transport and provided additional charge storage through the redox activity of PANI [39]. Qi et al. presented a novel approach for constructing advanced anode materials by fabricating Co_3_O_4_/polyaniline (PANI) core–shell arrays (CSAs). Using chemical bath deposition (CBD) followed by electrodeposition, they achieved a highly conductive and stress-buffering PANI shell intimately coating Co_3_O_4_ nanorods. The resulting Co_3_O_4_/PANI CSAs demonstrated significantly improved electrochemical performance, with a reversible capacity of 787 mAh g^−1^ and enhanced cycle stability compared to the unmodified Co_3_O_4_ counterpart (Figure 8a). The core–shell design, which merges the high conductivity of PANI with the energy storage capability of Co_3_O_4_, opens up new possibilities for fabricating inorganic–organic composite electrodes for energy storage [73].

Gu et al. tackled the major challenges in lithium-sulfur (Li-S) batteries, such as the shuttle effect of lithium polysulfides (LiPS), poor conductivity, and volume expansion during charge-discharge cycles, by designing CoFe_2_O_4_ nanotubes decorated with polyaniline (PANI). The CoFe_2_O_4_ nanotubes provided void space to accommodate sulfur and mitigate volume changes, while PANI enhanced the overall conductivity and LiPS retention. This smart design resulted in a S/CoFe_2_O_4_@PANI electrode with a high initial capacity of 864.1 mAh g^−1^ and a reversible capacity of 582.6 mAh g^−1^ after 500 cycles at 2 C, marking a significant improvement in cycling stability for Li-S batteries [74].

### 6.4. Factors Influencing the Electrochemical Performance of Batteries

Regarding the battery material, cobalt oxide/PANI nanocomposites can be affected by different factors that influence the electrochemical performance of the batteries [4], such as:

*Morphology and structural integrity*: The morphology of cobalt oxide/PANI composites, such as core–shell or nanofiber structures, influences ion diffusion and electron transport, directly impacting battery performance.

*Polymer content*: The content of PANI in the composite should be optimized to balance between providing sufficient conductivity and maintaining a high specific capacity. An optimal balance ensures enhanced electrochemical performance without compromising capacity.

*Interfacial interactions*: Strong interfacial interactions between cobalt oxide and PANI are essential for efficient charge transfer and maintaining structural integrity during cycling. Weak interactions can lead to detachment or degradation of the composite, adversely affecting performance.

## 7. Supercapattery: Basic Concept, Comparison, and How It Works

Supercapatteries are the hybrid energy storage devices that combine the properties of both supercapacitors and batteries. They aim to bridge the gap between these two technologies, offering the fast charge-discharge rates of supercapacitors and the high energy density typical of batteries.

*Driving force for its origin*: Supercapacitors are known for high power density, meaning they can deliver energy quickly, but they have relatively low energy storage capacity (energy density). Furthermore, batteries are known for their high energy density, meaning they store a large amount of energy, but their power density (ability to release energy quickly) is lower compared to supercapacitors. This has resulted in the goal to develop supercapatteries. Supercapatteries usually involve a hybrid structure, which may consist of battery-type and capacitive-type electrode materials:

*Battery-type materials*: Electrodes made from materials with faradaic (battery-like) behavior, such as transition metal oxides or conducting polymers.

*Capacitor-type materials*: Electrodes that use materials with electrostatic storage, such as carbon-based materials (graphene, activated carbon).

Characteristics of supercapatteries:

*Energy density*: Supercapatteries have higher energy density than traditional supercapacitors, closer to that of batteries.

*High Power Density*: They retain a higher power density, allowing for rapid charge and discharge, more typical of supercapacitors.

*Lifespan*: Like supercapacitors, supercapatteries have longer cycle lives than conventional batteries, as they can handle more charge-discharge cycles without significant degradation.

*Applications*: These devices are particularly useful in applications requiring both quick energy delivery and reasonable storage capacity, such as in electric vehicles, renewable energy systems, and portable electronics.

**Figure 9:**
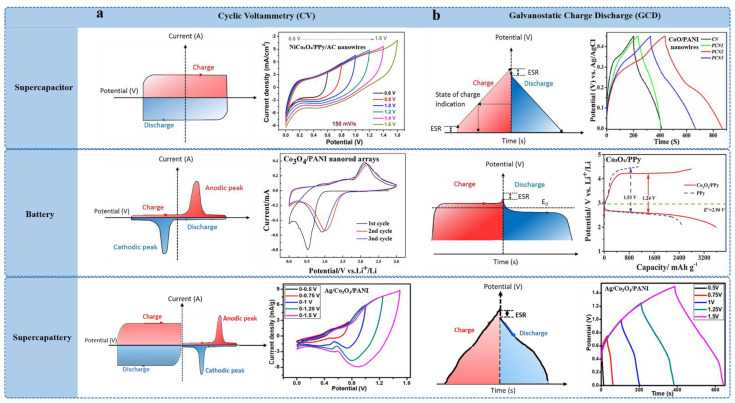
Comparative electrochemical differences in (**a**) CV and (**b**) GCD curves of cobalt oxide/conducting polymer (PPy and PANI)-based electrode materials for supercapacitor, battery, and supercapattery applications (ESR is equivalent series resistance) [1,49,53,69,71,73] (*reprinted with permission from* [1]; *copyright 2018 American Chemical Society*).

The electrochemical performance of energy storage devices like supercapacitors, batteries, and supercapatteries can be clearly distinguished by analyzing their cyclic voltammetry (CV) and galvanostatic charge-discharge (GCD) profiles. CV and GCD are used to assess the redox behavior and charge/discharge under constant current, respectively. The electrochemical difference between supercapacitors, batteries, and supercapatteries in terms of their CV and GCD curves are provided in Table 5 and Figure 9.

## 8. Cobalt Oxide/PPy and Cobalt Oxide/PANI Nanocomposites for Supercapatteries

### 8.1. Synthesis Strategies

The synthesis strategies for cobalt oxide nanocomposites with PPy and PANI for supercapatteries include:

*In-situ polymerization*: Pyrrole or aniline monomers are polymerized in the presence of pre-synthesized cobalt oxide nanoparticles, leading to the formation of the Co_3_O_4_/conducting polymer composite.

*Electrochemical deposition*: Cobalt oxide is electrodeposited onto a conductive substrate, followed by electropolymerization of pyrrole or aniline to form PPy or PANI on the cobalt oxide surface.

*Hydrothermal synthesis*: Cobalt oxide is synthesized via hydrothermal methods, and the conducting polymer is subsequently polymerized in-situ on the cobalt oxide.

*Sol-gel method with polymerization*: Cobalt oxide is synthesized using the sol-gel method, followed by in-situ oxidative polymerization of pyrrole or aniline to form the composite.

*Template-assisted synthesis*: A hard or soft template is used to create nanostructured cobalt oxide, followed by the deposition of a conducting polymer.

*Mechanical mixing and coating*: Pre-synthesized cobalt oxide nanoparticles are physically mixed with the conducting polymer, often followed by oxidative polymerization.

*Co-precipitation with pyrrole polymerization*: Co-precipitation of cobalt oxide is combined with the polymerization of pyrrole or aniline to form the nanocomposite.

### 8.2. Role of PPY and PANI in Enhancing Supercapattery Performance

PPy and PANI enhance the performance of cobalt-oxide-based supercapatteries in several ways:

*High Conductivity*: Cobalt oxide alone has moderate electrical conductivity. PPy and PANI, as conducting polymers, enhance the composite’s electrical conductivity, allowing for faster electron transport during charge-discharge processes. This improved conductivity increases the power density of the supercapattery.

Pseudocapacitance: PPy and PANI contribute to pseudocapacitance due to their ability to undergo fast and reversible redox reactions during charge-discharge. This pseudocapacitive behavior enhances the overall capacitance of the composite, boosting energy storage capacity.

Structural Flexibility: PPy and PANI provide mechanical flexibility to the composite, accommodating volume changes in cobalt oxide during cycling. This reduces structural degradation and enhances cycle stability.

Synergistic Effect: The combination of capacitive properties of PPy or PANI and cobalt oxide’s faradaic behavior results in a synergistic effect, improving both energy and power density.

### 8.3. Electrochemical Performance of Cobalt Oxide/PANI Nanocomposites in Supercapatteries

There are a limited number of studies exploring the interaction between cobalt oxide and conducting polymers, such as PPy and PANI, to form electrode materials for supercapattery applications. Addressing this research gap presents a valuable opportunity to make significant advancements in the fabrication of energy storage devices.

In a recent study on supercapatteries, Iqbal et al. fabricated silver (Ag)- and Co_3_O_4_-decorated PANI fibers by a combination of in-situ aniline oxidative polymerization and hydrothermal synthesis. The structural and morphological analyses revealed that PANI fibers were uniformly coated with Ag and Co_3_O_4_ nanograins, forming a fibrous nanocomposite with high purity and crystallinity. Electrochemical evaluations revealed that the incorporation of Ag and Co_3_O_4_ significantly enhanced the electrochemical properties of the PANI matrix (Figure 8b). The synergistic effect of these materials provided additional active sites for faradaic reactions, leading to a higher specific capacity. The Ag/Co_3_O_4_/PANI ternary nanocomposite exhibited a remarkable specific capacity of 262.62 C g^−1^ at a scan rate of 3 mV s^−1^. Furthermore, the material depicted high energy and power densities, reaching 14.01 Wh kg^−1^ and 165.00 W kg^−1^, respectively. The cycling stability of the supercapattery, consisting of Ag/Co_3_O_4_/PANI as the battery-type electrode and activated carbon as the counterpart, was notably impressive. The specific capacity initially increased during the first 1000 cycles and remained stable up to 2500 cycles. After 3500 cycles, the supercapattery retained 121.03% of its initial capacity, reflecting its excellent long-term stability (Figure 8c,d). This study underscores the potential of metal-doped Ag/Co_3_O_4_/PANI nanocomposites in advanced energy storage applications due to their enhanced electrochemical performance and durability [71].

Together, such studies highlight the importance of cobalt oxide in combination with conductive polymers like PANI in advancing battery and supercapacitor technologies. The core–shell structures, synergistic effects, and tailored composites are crucial for enhancing electrochemical performance, including capacity retention, conductivity, and cycle stability, making these materials highly promising for next-generation energy storage systems.

### 8.4. Factors Influencing the Electrochemical Performance of Supercapatteries

*Morphology of cobalt oxide*: The surface area, shape, and size of cobalt oxide nanostructures influence ion diffusion and electron transfer, directly affecting the overall performance. Nanostructures like nanowires or nanosheets offer a high surface area and improve charge storage.

*Loading amount of conducting polymer*: The amount of PPy or PANI influences the balance between capacitive and faradaic charge storage. Excess polymers may block active sites on cobalt oxide, while insufficient polymers reduce conductivity.

*Composite conductivity*: The overall conductivity of the composite impacts the charge transfer resistance and the charge-discharge rates. Highly conductive polymers and well-distributed cobalt oxide nanoparticles improve electron transport.

*Electrolyte choice*: The electrolyte determines the ion transport rate and the operating voltage window. Aqueous electrolytes (e.g., H_2_SO_4_ or KOH) provide fast ion transport, while organic electrolytes enable a higher voltage window but may limit ion mobility.

*Synthesis temperature*: High synthesis or annealing temperatures can enhance crystallinity and conductivity but may also degrade the polymer, reducing its performance.

*Cobalt oxide–polymer interaction*: Strong interfacial bonding between cobalt oxide and PPy ensures better charge transfer and structural stability, which is crucial for long-term cycling.

## 9. Effect of Electrolytes on Supercapacitors, Batteries, and Supercapatteries

Electrolytes play a crucial role in determining the overall performance of energy storage devices such as supercapacitors, batteries, and supercapatteries. They influence the ion transport, electrochemical stability, operating voltage window, and charge storage mechanism, all of which affect the device’s energy and power densities, cycle life, and efficiency. Table 6 illustrates the effects of electrolytes on the electrochemical performance of supercapacitors, batteries, and supercapatteries.

In supercapacitors, electrolytes facilitate the movement of ions between the electrodes during charging and discharging. The performance of a supercapacitor is highly dependent on the nature of the electrolyte, which impacts its specific capacitance, rate capability, and voltage range. Energy density is directly related to the voltage window (E = ½ CV^2^). Organic (e.g., TEABF_4_ in acetonitrile, propylene carbonate) and ionic liquid (e.g., EMIMBF_4_) electrolytes provide higher energy densities due to their wider voltage windows compared to aqueous (e.g., KOH, H_2_SO_4_, Na_2_SO) electrolytes. Aqueous electrolytes offer higher ionic conductivity, leading to faster ion movement and higher power density. Electrolytes’ chemical stability and reactivity with electrode materials impact the cycle life. Aqueous electrolytes may cause electrode corrosion over time, while organic and ionic liquids typically offer better long-term stability [75].

In batteries, electrolytes enable the movement of ions between the cathode and anode during charge and discharge. The choice of electrolyte significantly influences the ionic conductivity, chemical stability, and electrochemical window, which in turn affect the battery’s capacity, voltage, efficiency, and lifespan. The energy density of a battery depends on the capacity of the electrodes and the voltage window provided by the electrolyte. Liquid electrolytes (e.g., LiPF_6_ in organic solvents like EC, DMC) in lithium-ion batteries offer high voltage ranges (3.6–4.2 V), enabling high energy density. Solid-state electrolytes (e.g., Li_7_La_3_Zr_2_O_12_, sulfide-based solid electrolytes) enhance battery safety by reducing risks related to leakage and flammability. Additionally, they provide better stability against dendrite formation, which is critical in lithium-based systems. Additionally, for safety and stability concerns, polymer electrolytes, such as PEO-based electrolytes, are fabricated for their flexibility, light weight, and wearability, as they are safer than liquid electrolytes. Electrolytes’ chemical stability ensures minimal side reactions with electrodes, reducing capacity fading and improving cycling life. High-purity, stable electrolytes minimize self-discharge and internal resistance [75].

Supercapatteries combine the characteristics of both supercapacitors and batteries, and the choice of electrolyte has a profound impact on their hybrid performance. The electrolyte must balance high energy density (from battery-like faradaic processes) and high power density (from capacitor-like electrostatic processes). The electrolyte determines how well the supercapattery can balance energy density (from battery-like electrodes) and power density (from supercapacitor-like electrodes). Organic (e.g., LiPF_6_ in organic solvents) and ionic liquid electrolytes offer higher energy density, but aqueous electrolytes provide superior power density due to faster ion transport. Electrolyte stability is critical for maintaining long-term cycle life in supercapatteries. Organic electrolytes offer better voltage stability, while aqueous electrolytes, though faster, may cause electrode corrosion over extended cycling. Ionic liquid electrolytes provide excellent stability but can suffer from poor rate capability [75].

## 10. Other Miscellaneous Electrochemical Studies and Applications

With the individual combination of cobalt oxide, PPy and PANI, there are a plethora of studies which state their application in energy-related fields such as electrocatalysis, photocatalytic degradation of dyes, sensing, and so on. A few of them are mentioned in Table 7.

## 11. Current Challenges

Despite significant advancements in cobalt-oxide-based nanomaterials and their hybrids with conductive polymers for energy storage applications such as supercapacitors, batteries, and supercapatteries, several critical challenges remain. These challenges must be addressed to fully harness their potential in high-performance electrochemical devices.

*Agglomeration and structural degradation*

One of the foremost challenges faced by cobalt-oxide-based nanomaterials is the agglomeration of low-dimensional nanostructures. Agglomeration leads to a reduction in the active surface area, diminishing the number of available electrochemically active sites. Moreover, repeated charge-discharge cycles cause mechanical stress, leading to structural pulverization and cracks in the electrode materials. This degradation is a key factor in the rapid decay of performance in supercapacitors and batteries, particularly under long-term cycling.

*Synthesis and material uniformity*

While numerous synthesis methods for cobalt-oxide-based nanostructures have been developed, achieving reproducible, property-on-demand materials remains a major challenge. The trade-off between uniformity, size control, and large-scale production complicates the design of cobalt oxide nanomaterials with consistent electrochemical properties. This variability can lead to uneven electrochemical performance, making it difficult to optimize for large-scale practical applications.

*Side reactions in electrochemical devices*

Cobalt-oxide-based materials are prone to side reactions when used in electrochemical energy storage devices, which can negatively impact performance. In real-world systems, factors such as electrode–electrolyte interactions, the stability of the electrolyte, and electrode degradation contribute to undesirable side reactions. These side reactions can lead to irreversible capacity loss and affect the overall efficiency of the system, particularly in the complex operating conditions of supercapacitors, batteries, and hybrid devices like supercapatteries.

*Polymer degradation in hybrids*

The integration of cobalt oxide with conductive polymers such as PANI or PPy has shown promise due to the enhanced redox reactions and conductivity these hybrids offer. However, one of the main issues with these polymer-based nanostructures is their mechanical degradation over extended cycling. Conductive polymers often suffer from volumetric expansion and contraction during the charge-discharge process, leading to cracks and a reduction in mechanical integrity. This degradation causes a loss in electrochemical performance, especially in long-term cycling, limiting the lifespan of energy storage devices.

*Electrochemical pathways and reaction mechanisms*

Although there have been significant efforts to understand the reaction mechanisms of cobalt oxide during ion storage and electrocatalysis, the exact pathways remain incompletely understood. Electrochemical reactions are complex, and multiple variables—such as the type of electrolyte, separator, and external environmental conditions—affect performance. Continued theoretical and experimental studies are required to uncover the detailed mechanisms governing these materials to improve their performance predictability and optimize their electrochemical behavior.

## 12. Future Outlook

Significant strides have been made in the development of cobalt oxide/conducting polymer nanocomposites for energy storage devices like supercapacitors, batteries, and supercapatteries. These materials demonstrate impressive electrochemical properties, including high capacitance, energy density, and conductivity. However, overcoming current limitations will lead to even broader practical applications. Future work is likely to focus on several key areas:

*Deepening understanding of reaction mechanisms*: Continued exploration of the fundamental electrochemical processes, especially the interaction between cobalt oxides and conducting polymers, will enhance the stability and efficiency of these materials. This includes studying ion transport, charge transfer, and the redox reactions that occur during cycling.

*Advanced material design*: The next phase of development will likely focus on the rational design of novel cobalt oxide/polymer hybrids. The integration of theoretical modeling and simulations will aid in predicting structure–property relationships, which is essential for optimizing the performance of these materials in energy storage systems. Simulations can guide the fabrication of hybrid structures with controlled porosity, particle size, and conductivity.

*Enhancing durability and conductivity*: The development of composites that incorporate reinforcing agents like carbon nanotubes, graphene, or metallic nanoparticles (e.g., silver) will address issues of mechanical degradation and enhance conductivity. Such hybrid materials could offer better cycling stability and prevent the volume expansion that often plagues cobalt oxide/polymer systems.

*Innovative synthesis techniques*: To facilitate large-scale commercialization, scalable and eco-friendly synthesis methods such as hydrothermal, electrochemical deposition, and sol-gel techniques will be further refined. These processes will enable the mass production of uniform cobalt oxide/polymer nanostructures with consistent electrochemical performance.

*Integration with next-gen devices*: The application of cobalt oxide/conducting polymer nanocomposites is poised to expand beyond traditional supercapacitors and batteries. With innovations in device architecture, such materials may play a key role in next-generation energy storage systems, such as flexible and wearable electronics or hybrid supercapatteries that bridge the gap between capacitors and batteries.

*Promising breakthrough*: Minimizing side reactions and their mechanisms could enhance the electrochemical performance of the electrode material.

Overall, future research will be geared towards optimizing the stability, conductivity, and scalability of cobalt oxide/conducting polymer hybrids. As theoretical understanding grows and synthesis techniques improve, these materials are expected to become central to the advancement of high-performance energy storage technologies.

## 13. Critical Thinking Insights and Recommendations

As electrode material for supercapacitors, the combination of cobalt oxide’s pseudocapacitive behavior with the high conductivity of PPy/PANI enhances energy storage, but challenges remain in optimizing cycling stability. For batteries, cobalt oxide’s high theoretical capacity pairs well with PPy/PANI’s flexibility, but side reactions and poor long-term stability need further investigation. For supercapatteries, the hybrid device benefits from cobalt oxide’s energy density and PPy/PANI’s fast charge transport, though achieving a balance between energy and power density is still a key challenge.

The critical analysis revealed that the focus should be on minimizing side reactions, enhancing cycling stability, and resolving conductivity challenges in cobalt oxide/PPy and cobalt oxide/PANI systems. The strength signifies that the cobalt oxide combined with PPy/PANI offers high energy and power densities with good flexibility, while the limitations include poor long-term stability, side reactions, and suboptimal performance at high rates.

Our unique perspective emphasizes the role of hybrid mechanisms in supercapatteries as a novel and underexplored area, offering insights into potential breakthroughs in energy density improvements. Furthermore, we recommend exploring advanced synthesis methods to improve interface stability and develop more robust electrolytes to mitigate side reactions.

## 14. Conclusions

In summary, cobalt-oxide-based nanocomposites, particularly those incorporating conducting polymers like polypyrrole (PPy) and polyaniline (PANI), hold great promise as high-performance electrode materials for supercapacitors, batteries, and supercapatteries. These materials benefit from the complementary properties of cobalt oxide’s excellent capacitance and the conductive polymers’ enhanced electron transport and pseudocapacitive behavior. Factors like morphology optimization, interfacial bonding, and controlled porosity significantly influence their performance in energy storage. While advances have been made, further exploration is needed, especially for battery applications, to unlock their full potential and ensure scalability for practical use in next-generation energy devices.

## Figures and Tables

**Table 2:** General differences between supercapacitors, batteries, and supercapatteries.

Parameter	Supercapacitors	Batteries	Supercapatteries
Energy storage mechanism	Electrostatic (capacitive)	Electrochemical (faradaic)	Hybrid: capacitive + faradaic reactions
Energy density	Low (1–10 Wh/kg)	High (100–265 Wh/kg, for LIB)	Moderate (between supercapacitors and batteries, 20–100 Wh/kg)
Power density	Very High (>10,000 W/kg)	Moderate to Low (200–2000 W/kg)	High (closer to supercapacitors, 1000–10,000 W/kg)
Charge-discharge speed	Very fast (seconds to minutes)	Slow (hours)	Fast (minutes to tens of minutes)
Cycle life	Long (up to 1,000,000 GCD cycles)	Shorter (500–3000 cycles)	Long (10,000–100,000 cycles)
Voltage window	2.5–2.7 V per cell	3.6–4.2 V per cell	2.5–4.0 V (material dependent)
Response time	Instantaneous	Slow	Fast (intermediate between the two)
Self-discharge	Quickly when not in use	Low	Moderate (lower than supercapacitors but higher than batteries)
Cost	Lower per unit of power	Higher per unit of energy	Moderate
Application	High-power applications (e.g., regenerative braking)	Energy storage applications (e.g., mobile phones, EVs)	Hybrid applications (e.g., electric vehicles, power grids)

**Table 5:** Comparison of CV and GCD of supercapacitors, batteries, and supercapatteries.

Device	CV	GCD
**Supercapacitors**		
EDLCs	Rectangular	Linear (symmetric)
Pseudocapacitors	Oval with redox peaks	Slightly non-linear (asymmetric)
Hybrid capacitors	Rectangular with redox peaks (mixed)	Combination of linear and non-linear
**Batteries**	Sharp redox peaks	Non-linear with distinct plateaus
**Supercapatteries**	Quasi-rectangular with broad redox peaks	Quasi-linear with slight curvature

**Table 6:** Effects of electrolytes on the electrochemical performance of supercapacitors, batteries, and supercapatteries.

Parameter	Supercapacitors	Batteries	Supercapatteries
Energy density	Higher with organic and ionic liquids; lower with aqueous electrolytes	Higher in organic/solid electrolytes, liquid electrolytes (e.g., lithium-ion systems)	Balanced; depends on electrolyte selection (aqueous for power, organic for energy)
Power density	Higher with aqueous electrolytes due to fast ion transport	Moderate to low due to solid-state diffusion limits	Higher with aqueous electrolytes; organic provides balanced performance
Cycle life	Aqueous and ionic liquids can provide excellent stability; organic electrolytes have moderate stability	Longer with solid-state or stable liquid electrolytes; dendrite formation is a risk with liquid	Long-term stability depends on electrolyte–electrode compatibility
Rate capability	Aqueous electrolytes provide superior rate capability	Lower in solid-state, moderate in liquid systems	Aqueous electrolytes provide faster charge-discharge; organic is slower
Safety	Aqueous and ionic electrolytes are safer (non-flammable)	Solid-state offers better safety than liquid; organic is flammable	Solid-state or aqueous electrolytes offer enhanced safety

**Table 7:** Miscellaneous combinations of cobalt oxide, PPy, and PANI for different electrochemical studies and their applications.

Composite	Cobalt as	Polymer	Material	Application	Ref.
Co–iron oxide/PANI (CFOP)	Co metal	PANI	Separator	Li-S batteries	[76]
PPN-CoO	CoO	PPy	Anode	Electrocatalytic water splitting	[77]
Co_3_O_4_/Ppy/MWCNT	Co_3_O_4_	PPy	Electrode	Electrocatalytic water splitting	[78]
ChGP/Co_3_O_4_	Co_3_O_4_	Chitosan, PANI	Nanocomposite	Photocatalytic dye degradation	[79]
Co_3_O_4_/PVA/PVP	Co_3_O_4_	Poly(vinyl alcohol), poly(vinyl pyrolidone)	Nanocomposite	Supercapacitor	[80]
NiCo_2_O_4_/PVA	CoO	Poly(vinyl alcohol)	Electrode	Supercapacitor	[81]
LiCoO_2_ (MLCO)	CoO	Poly[N,N-bis(2-cryano-ethyl)-acrylamide]	Cathode	Li-ion batteries	[82]
[Co(tfbdc)(4,40-bpy)(H2O)2] Co-LCP	Co metal	Coordination polymer	Anode	Li-ion batteries	[83]
PDs-CoO	CoO	Polyethylene glycol diacid	Nanocomposite	Supercapacitor	[84]
Mg/SPE/Co_3_O_4_	Co_3_O_4_	Methyl cellulose	Electrolyte films	Batteries	[85]
Phosphene-PANI	-	PANI	Cathode	Zn-ion batteries	[72]
Co_3_O_4_/CoO/Co/C	Co_3_O_4_, CoO, Co metal	-	Electrode	Li-ion batteries, supercapacitors, and OER	[86]
rGO/Co_3_O_4_/Ag/ activated carbon	Co_3_O_4_	-	Anode	Supercapatteries	[87]
Co/C_2_N	Cobalt oxide	C_2_N network	Catalyst	HER	[88]

## Data Availability

No other data are available for this work.
